# See Me, Hear Me: Racial Discrimination Among Women Seeking Breast Cancer Care

**DOI:** 10.21203/rs.3.rs-4837604/v1

**Published:** 2024-09-20

**Authors:** Naomi Ko, Lauren Oshry, Ruth Lederman, Haley Gagnon, Tsion Fikre, Daniel Gundersen, Anna Revette, Ashley Odai-Afotey, Olga Kantor, Dawn Hershman, Katherine Crew, Nancy Keating, Rachel Freedman

**Affiliations:** Boston University School of Medicine/Boston Medical Center; Boston University School of Medicine/Boston Medical Center; Dana-Farber Cancer Institute; Dana-Farber Cancer Institute; Boston University School of Medicine/Boston Medical Center; Dana-Farber Cancer Institute; Dana-Farber Cancer Institute; Dana-Farber Cancer Institute; Brigham and Women’s Hospital; Brigham and Women’s Hospital; Columbia University Irving Medical Center, Hervert Irving Comprehensive Cancer Center; Columbia University; Brigham and Women’s Hospital; Harvard Medical School; Dana Farber Cancer Institute

## Abstract

Discrimination can contribute to worse health outcomes, but its prevalence in breast cancer is not well studied. We aimed to understand how women with stage I-III breast cancer faced discrimination in health care and everyday settings through a cross-sectional survey. 296 women, 178 (60%) Non-Hispanic White (NHW), 76 (26%) Non-Hispanic Black (NHB), and 42 (14%) Hispanic participated. NHB women reported significantly more discrimination in everyday life compared to NHW women (score 20.1 vs 16.1, p<.001) and Hispanic women (score 20.1 vs 16.0, p<.001). In the health care setting, NHB had statistically more frequent reports of being ignored (23.7% vs. 5.6%), treated with less respect (21.1% vs. 7.3%), and treated with less courtesy (18.7% vs. 6.2%; all P=<.001) when compared to NHW women. NHB women experience a higher degree of discrimination both inside and outside of health care. Further research to understand discrimination on breast cancer outcomes is warranted.

## Introduction

Despite advances in prevention, early detection, and treatment, racial inequities in breast cancer persist. Black women experience a 40% higher breast cancer-specific mortality than White women [1, 2]. The reasons for this inequity are complex, involving tumor characteristics, social determinants of health, and treatment-related factors [3]. Among all the factors, discrimination can play an insidious role because it has the potential to corrode trust and negatively impact decision making, thereby affecting cancer outcomes [4]. In addition, discrimination may mediate genomic, epigenetic, and physiologic alterations and impact access, receipt, completion, and tolerability of treatment, all of which may contribute to disparities in breast cancer outcomes [5, 6]. Compelling research in recent years has clearly demonstrated the ongoing damage of structural, cultural and individual-level racism present across multiple facets of the health care system, including societal, institutional, and clinical treatment settings [7, 8, 9].

Previous studies have documented higher rates of perceived discrimination among Black women diagnosed with breast cancer compared to White women [10, 11]. However, details on the setting and type of discrimination are lacking, with limited information available on the experiences among those treated for cancer. Our research team administered a survey to a racially and ethnically diverse population of women who had undergone treatment for breast cancer in New York and Boston. As part of this study, we sought to understand experiences of discrimination within and outside of health care settings. We also examined how experiences with discrimination may impact treatment receipt.

## Patients and Methods

### Study overview and population

We invited adult women diagnosed with a history of stage I-III breast cancer during 2013–2017 to participate in a one-time interviewer-administered survey. Interviews were conducted during 2018–2020. The survey instrument included questions on demographics, treatment decisions, treatment receipt, breast cancer knowledge, experiences with discrimination, and potential barriers to treatment receipt. We recruited a diverse population of non-Hispanic White (hereafter, White), non-Hispanic Black (hereafter, Black), and Hispanic participants. Participants had to understand and speak English or Spanish and had to receive some or all of their cancer care (at least 3 visits) at a participating center (Dana-Farber Cancer Institute, Boston Medical Center [both in Boston, MA] or Columbia University Irving Medical Center [New York, NY]). The Institutional Review Board of each participating center approved the study, and the study conformed to the standards set forth in the Declaration of Helsinki. Additional details of the study methodology have been previously published [12, 13].

### Survey Instrument

The survey used for this analysis included questions about discrimination derived from the validated Everyday Discrimination Scale [14, 15, 16]. To examine experiences in the health care setting, participants were asked to consider the same questions but instead, as related to their experiences within the health care setting. For all questions, participants characterized their experiences as occurring either “never,” “rarely,” “sometimes,” “often,” or “always.” For each component of potential adjuvant treatment, participants were asked about whether it was recommended to them *and* if they initiated it, including radiation, chemotherapy, hormonal therapy, and trastuzumab.

### Statistical Analysis

First, we compared patient characteristics by race and ethnicity using Chi square and Fisher’s exact tests. We then calculated the summary experience of discrimination scores separately for discrimination in everyday life and in the health care setting by summing responses to each item (scored 1 to 5 for ‘never’ to ‘always’). Within each race and ethnicity group, differences in discrimination scores between the everyday setting and the health care setting were compared through paired-sample t-tests. For both the everyday setting and the health care setting, discrimination scores were compared across race and ethnicity groups through one-factor analysis of variance followed by Tukey’s pairwise comparisons. Responses to individual items from the discrimination scales were dichotomized as “never/rarely” and “sometimes/often/always” and Chi-square testing was used to compare the percent responding ‘sometimes/often/always’ across race and ethnicity groups. Because non-response to questions was infrequent (n = 3 participants left some discrimination questions blank), these participants were excluded from the analysis. To test for reliability of the two discrimination measures, we performed Cronbach’s alpha testing for each discrimination scale. Finally, we used Chi-square testing to evaluate univariate associations of responses to the discrimination scales and treatment receipt. Of note, because race itself wasn’t associated with treatment receipt in prior analyses [12] we focused on discrimination for these potential associations.

## Results

Overall, 296 women, 178 (60%) White, 76 (26%) Black, 42 (14%) Hispanic, completed interviews. Details on response rates have been published previously [12]. Table 1 displays summary patient characteristics. Most had hormone receptor-positive breast cancer and stage I-II disease at diagnosis. Black and Hispanic (vs. White) women reported lower educational attainment, and Black women were younger than other sub-groups at the time of interview.

Table 2 shows the summary scores for everyday and heath care settings by race and ethnicity. Within each race and ethnicity group, discrimination summary scores were significantly lower in the health care setting compared with everyday life (paired sample t-tests, p<0.001 for White vs. Hispanic women, p<0.002 for White vs. Black women). In the everyday life setting, discrimination scores were higher for Black (mean 20, range 10–43) compared to White women (mean 16 range 10–33; p<0.001) and Hispanic women (mean 16 range 10–33; p<0.001). There was no significant difference in mean everyday discrimination score for Hispanic vs. White women (p=0.996). In the health care setting, mean discrimination scores for Black women (mean 15, range 10–44) were significantly higher than those for White women (mean 13, range 10–25, p=0.008) and borderline significantly higher than Hispanic women (mean 13 range 10–21, p=0.056). There were no significant differences in mean discrimination scores for Hispanic vs. White women in the health care setting (p=0.985).

Individual survey question responses regarding discrimination in everyday life experiences and within the health care setting are displayed in Tables 3 and 4, respectively. When compared to White women, Black women experienced significantly more discrimination in many areas. In the everyday setting, Black women experienced significantly more discrimination on 9 of the 10 items from the instrument. Further, Black women experienced significantly more discrimination than Hispanic women for 7 of the 10 items. In contrast, Hispanic women experienced significantly higher discrimination in the everyday setting than White women on only 1 of the 10 items from the instrument (‘being threatened/harassed’).

While less discrimination was reported in the health care setting overall, Black women still experienced more discrimination than White women on 6 of the 9 items from the instrument (‘treated with less courtesy’, ‘treated with less respect’, ‘people act as if they think you are not smart’, ‘people act as if they are afraid of you’, ‘people act as if they think you are dishonest’, and ‘people ignore you or act as if you are not there’). Hispanic women reported higher discrimination than White women on only 1 item (‘treated with less courtesy’), and lower discrimination than White women on 1 item (‘people act as if they are better than you’). Black women reported significantly higher discrimination than Hispanic women on 1 item (‘people act as if they are better than you). Cronbach coefficients for the Everyday Discrimination scale and the discrimination within health care scales were 0.89 and 0.87, respectively, indicating a high degree of reliability.

In univariate analyses of discrimination item responses and treatment receipt (Table 5), only one item in the everyday discrimination scale (people act as if they think you are dishonest) was significantly associated with treatment; participants reporting this item as sometimes/often/always being less likely to receive recommended treatments. With regard to responses for the discrimination within health care measure, responses of sometimes/often/always for four items (treated with less courtesy, treated with less respect, people act as if you are not smart, people act as if they think you are dishonest) were significantly associated with less treatment receipt (Table 5).

## Discussion

Within this cross-sectional survey of diverse breast cancer survivors, we report the stark difference in experiences of discrimination between NH White, Black, and Hispanic women treated for breast cancer in everyday life and the health care setting. To our knowledge, this is the first study to both confirm that Black women report discrimination significantly more often than other women, and to detail the setting and situation where the discrimination is felt in the context of a cancer diagnosis. Our measures demonstrated a high degree of reliability for the experiences reported within and outside of health care. And, despite small sample sizes for those declining at least one component of cancer-directed therapy, we observed several significant associations for discrimination and treatment receipt. Our results add to the growing body of literature aimed to better understand the prevalence and potential impact of discrimination, particularly in the health care, and more specifically cancer care, setting.

Multiple publications have previously demonstrated strong evidence for the impact of structural racism on health, not only when systemic racism occurs within health care but also because of the substantial impact of factors such as neighborhood segregation and public policy [5–8]. Less attention has been paid to the individual, day-to-day experiences for patients with cancer and how this may contribute to health outcomes. Other investigators, such as Sutton *et al.*, have reported a greater incidence of Black women experiencing discrimination in the health care setting compared to White women (47% vs 16% respectively) and a high incidence of lifetime discrimination (82% of NH Black women vs 19% of NH White women) [10, 11]. However, these previous reports have included women who are largely privately insured and college educated, or participants in a single health care system [10, 11]. Our participants were less likely to have private insurance (63%) or a college degree (33.6%), and they were treated at three separate health care systems in two states. Thus, our findings may be more representative of the demographics for Black women with breast cancer.

Our data demonstrate that overall rates of self-reported discrimination in everyday life were significantly higher than in the health care setting and self-reported discrimination among Black women was significantly higher than white women in both everyday and health care settings. Everyday discrimination is a serious concern underscoring challenges that women of color experience in their day-to-day lives. Emerging research on cancer inequities has begun to rigorously investigate the multiple consequences of racism, discrimination, and the chronic stress experienced by the most vulnerable populations. The burgeoning scientific study of living amongst these racist and discriminatory conditions chronically can lead to worse health outcomes is a rapidly growing and important field of research. Discrimination has been associated with lower quality of life among breast cancer survivors [11]. In addition, individual discrimination is associated with physiologic, behavioral and health care use responses that collectively adversely impact health outcomes [9]. Downstream negative consequences include but are not limited to medical mistrust, poor communication, delayed access to care, chronic inflammation, and allostatic load or weathering [6, 9, 17]. As our societal awareness and understanding of these challenges expand, future interventions to alleviate, reverse and mitigate these cancer inequities is critical.

Our results also identify *specific* experiences of Black patients, which can be addressed with thoughtful interventions and change in health care practices or protocols. For example, rates of reporting feeling ignored in the health care setting were twice as high for Black women than Hispanic women and four times higher than White women. In addition, Black women had higher rates of reporting being treated with less courtesy and less respect. These results can guide intentional changes within the health care setting to address these perceptions. Reinforcement of respect and courtesy can be a crucial first step and a collective priority.

Limitations of the study include the small sample sizes for some analyses and the focus on care in three systems only. The study adapted the discrimination scale which has not been validated in the health care setting. Further, patients who chose to participate in the study may be favorably predisposed to the health care system because they had engaged in cancer care at the participating center, potentially skewing the results.

Overall, this study contributes to foundational work necessary to understand and alleviate discrimination experienced by Black women receiving treatment for breast cancer. An awareness of the frequency and scope of discrimination is an essential step to remedying the problem. Immediate efforts can focus on identifying and mitigating factors contributing to specific experiences of discrimination in the healthcare setting including Black women feeling unseen and unheard, feeling treated with less courtesy and respect, and feeling as though people think they are dishonest or not smart.

## Figures and Tables

**Table 1.** Participant characteristics (N, %)

Characteristic	Overall (*n* = 296)	Non-Hispanic White (*n* = 178)	Non-Hispanic Black or African American (*n* = 74)	Hispanic (*n* = 44)	*p*-value
*Age at time of interview, years (n = 294)*					
≤ 50	107 (36.3)	53 (29.9)	33 (44.6)	21 (47.7)	0.004†
50–60	94 (31.9)	53 (29.9)	25 (33.8)	16 (36.4)	
≥ 61	94 (31.9)	71 (40.1)	16 (21.6)	7 (15.9)	
*Tumor subtype at diagnosis*					
ER+ HER2-	211 (71.3)	136 (76.4)	45 (60.8)	30 (68.2)	0.14‡
ER+HER2 +	24 (8.1)	9 (5.1)	9 (12.6)	6 (11.4)	
ER- HER2 +	9 (3.0)	5 (2.8)	2 (2.7)	2 (4.6)	
Triple Negative	43 (14.5)	22 (12.4)	16 (21.6)	5 (11.4)	
Undetermined	9 (3.0)	6 (3.4)	2 (2.7)	1 (2.3)	
*Stage at diagnosis (n = 294)*					
I	169 (57.5)	111 (62.7)	35 (48.0)	23 (52.3)	0.02‡
II	98 (33.3)	56 (31.6)	27 (37.0)	15 (34.1)	
III	24 (8.2)	7 (4.0)	11 (15.1)	6 (13.6)	
DCIS	3 (1.0)	3 (1.7)	0 (0.0%)	0 (0.0)	
*Highest educational attainment (n = 294)*					
≤ 9^th^ grade	19 (6.5)	3 (1.7)	10 (13.5)	6 (14.3)	< 0.001 †
High school grad / some college	112 (38.1)	59 (33.2)	37 (50.0)	16 (38.1)	
College graduate	65 (22.1)	41 (23.0)	18 (24.3)	6 (14.3)	
Additional courses / advanced degree	98 (33.3)	75 (42.1)	9 (12.2)	14 (33.3)	
*Marital status* (n=295)^**^					
Married	178 (60.3)	122 (68.5)	32 (43.2)	24 (55.8)	0.0007†
Not married	117 (39.7)	56 (31.5)	42 (56.8)	19 (44.2)	
Health Insurance before diagnosis					< 0.001‡
Commercial/HMO	188 (63.5)	124 (70.0)	36 (48.7)	28 (63.6)	
Medicare	55 (18.6)	39 (21.9)	15 (20.3)	1 (2.3)	
Medicaid	47 (15.9)	14 (7.9)	21 (28.4)	12 (27.3)	
None	6 (2.0)	1 (0.6)	2 (2.7)	3 (6.8)	
*Chemotherapy*					
Treatment recommended*	152 (51.4)	76 (42.7)	50 (67.6)	26 (59.1)	0.002‡
Treatment received	146 (96.1)	74 (97.4)	48 (96.0)	24 (92.3)	0.39‡
*Radiation*					
Treatment recommendedˆ	228 (77.0)	127 (71.4)	67 (90.5)	34 (77.3)	0.009‡
Treatment received	220 (96.5)	124 (97.6)	63 (94.0)	33 (97.1)	0.36‡
*Endocrine Therapy*					
Treatment recommended	228 (77.0)	139 (78.1)	53 (71.6)	36 (81.8)	0.39 †
Treatment received	222 (97.4)	133 (95.7)	53 (100.0)	36 (100.0)	0.23‡
*Trastuzumab*					
Treatment recommended +	31 (10.5)	14 (7.9)	12 (16.2)	5 (11.4)	0.02‡
Treatment received	29 (93.6)	14 (100.0)	10 (83.3)	5 (100.0)	0.29‡
*Treatment(s) declined* (*n* = 21)					
Declined any component of recommended therapy	21 (7.1%)	11 (6.2%)	7 (9.5%)	3 (6.8%)	0.65†

† Chi-square p-value

‡ Fisher’s *p*-value

** One patient did not respond

* One patient reported being unsure if she had been recommended chemotherapy.

ˆ Two patients reported being unsure if they had been recommended radiation.

+ Twelve patients reported being unsure if they had been recommended trastuzumab.

Abbreviations: DCIS, ductal carcinoma in situ; ER, estrogen receptor; HER2, human epidermal growth factor receptor 2

**Table 2:** Summary scores of discrimination surveys by race and ethnicity

Discrimination experienced in the everyday setting						
	N	Mean	Std. dev	Min	Max	Pairwise comparison*
NH White	178	16.1	5.1	10	33	Reference group
NH Black+	76	20.1	7.6	10	43	p<.001
Hispanic	42	16.0	5.7	10	33	p=0.996

Overall table: p<0.001 from overall ANOVA comparing 3 means across groups

* p-value from Tukey's multiple comparison procedure

+ p=0.001 from Tukey's comparison of mean for NH Black vs. Hispanic women

**Table:** 

Discrimination in the health care setting						
	N	Mean	Std. dev	Min	Max	Pairwise comparison*
NH White	178	13.3	3.9	10	25	Reference group
NH Black+	76	15.3	6.9	10	44	p=.008
Hispanic	42	13.1	3.9	10	21	p=0.985

Overall table: p=0.007 from overall ANOVA comparing 3 means across groups

* p-value from Tukey's multiple comparison procedure

+ p=0.056 from Tukey's comparison of mean for NH Black vs. Hispanic women

**Table 3.** Responses for sometimes/often/always by race and ethnicity in the everyday setting

Survey Question	Race and Ethnicity (N, %)		
	NH White	NH Black	Hispanic
Treated with less courtesy	35 (19.7%)	30 (39.5%)***	8 (19.1%)
Treated with less respect	36 (20.3%)	30 (39.5%)***	8 (19.1%)
Receive poorer service at restaurants and in stores	16 (9.0%)	26 (34.7%)***	6 (14.3%)
People act as if they think you are not smart	30 (17.1%)	30 (40.0%)***	12 (28.6%)
People act as if they are afraid of you	23 (13.0%)	25 (33.3%)***	4 (9.5%)
People act as if they think you are dishonest	5 (2.8%)	18 (24.3%)***	1 (2.4%)
People act as if they are better than you	72 (40.7%)	45 (59.2%)***	15 (35.7%)
People ignore you or act as if you are not there	28 (15.8%)	26 (34.2%)***	7 (16.7%)
You are called names or insulted	15 (8.5%)	14 (18.4%)**	5 (11.9%)
You are threatened/harassed	14 (7.9%)	12 (15.8%)	8 (19.1%)**

Testing NH Black vs NH White and Hispanic vs NH White

* p<.05

** p<.01

*** p<.001

**Table 4.** Responding sometimes/often/always by race and ethnicity in a Health care Setting

Survey Question	Race and Ethnicity (N, %)		
	NH White	NH Black	Hispanic
Treated with less courtesy	11 (6.2%)	14 (18.7%)***	7 (16.7%) **
Treated with less respect	13 (7.3%)	16 (21.1%)***	5 (11.9%)
People act as if they think you are not smart	24 (13.5%)	18 (23.7%)**	4 (9.5%)
People act as if they are afraid of you	7 (3.9%)	8 (10.5%)**	1 (2.4%)
People act as if they think you are dishonest	3 (1.7%)	8 (10.5%)***	1 (2.4%)
People act as if they are better than you	42 (23.6%)	21 (27.6%)	2 (4.8%)**
People ignore you or act as if you are not there	10 (5.6%)	18 (23.7%)***	5 (11.9%)
You are called names or insulted	4 (2.3%)	4 (5.3%)	1 (2.4%)
You are threatened/harassed	2 (1.1%)	2 (2.6%)	1 (2.4%)

Testing NH Black vs NH White and Hispanic vs NH White

* p<.05

** p<.01

*** p<.001

**Table 5.** Item responses for discrimination scales and associations with treatment receipt

Discrimination scale question	Treatment receipt*		
*EVERYDAY DISCRIMINATION SCALE*			
	Yes N (%)	No N (%)	P value+
Treated with less courtesy:			P=.186
Never/rarely	209 (76.3)	14 (63.4)	
Sometimes/often/always	65 (23.7)	8 (36.4)	
Treated with less respect			P=.798
Never/rarely	206 (75.2)	16 (72.7)	
Sometimes/often/always	68 (24.8)	6 (27.3)	
Receive poorer service at restaurants and in stores			P=.144
Never/rarely	232 (84.7)	16 (72.7)	
Sometimes/often/always	42 (15.3)	6 (27.3)	
People act as if they think you are not smart			P=.395
Never/rarely	209 (76.3)	15 (68.2)	
Sometimes/often/always	65 (23.7)	7(31.8)	
People act as if they are afraid of you			P=.214
Never/rarely	228 (83.2)	16 (72.7)	
Sometimes/often/always	46 (16.8)	6 (27.3)	
People act as if they think you are dishonest			**P=.009**
Never/rarely	255 (93.1)	17 (77.3)	
Sometimes/often/always	19 (6.9)	5 (22.7)	
People act as if they are better than you			P=.596
Never/rarely	153 (55.8)	11 (50.0)	
Sometimes/often/always	121 (44.2)	11 (50.0)	
People ignore you or act as if you are not there:			P=.402
Never/rarely	216 (78.8)	19 (86.4)	
Sometimes/often/always	58 (21.2)	3 (13.6)	
You are called names or insulted			P=.742
Never/rarely	243 (88.7)	19 (86.4)	
Sometimes/often/always	31 (11.3)	3 (13.6)	
You are threatened/harassed			P=.289
Never/rarely	241 (87.9)	21 (95.5)	
Sometimes/often/always	33 (12.0)	1 (4.6)	
*DISCRIMINATION WITHIN HEALTH CARE SETTING*			
Treated with less courtesy			**P=.01**
Never/rarely	248 (90.5)	16 (72.7)	
Sometimes/often/always	26 (9.5)	6 (27.3)	
Treated with less respect	246 (89.8)	16 (72,7)	**P=.016**
Never/rarely			
Sometimes/often/always	28 (10.2)	6 (27.3)	
People act as if they think you are not smart			**P=.029**
Never/rarely	235 (85.8)	15 (68.2)	
Sometimes/often/always	39 (14.2)	7(31.8)	
People act as if they are afraid of you			P=.853
Never/rarely	259 (94.5)	21 (95.5)	
Sometimes/often/always	15 (5.5)	1 (4.6)	
People act as if they think you are dishonest			**P=.018**
Never/rarely	265 (96.7)	19 (86.4)	
Sometimes/often/always	9 (3.3)	3 (13.6)	
People act as if they are better than you			P=.246
Never/rarely	216 (78.8)	15 (68.2)	
Sometimes/often/always	58 (21.2)	7(31.8)	
People ignore you or act as if you are not there:			P=.073
Never/rarely	246 (89.8)	17 (77,3)	
Sometimes/often/always	28 (10.2)	5 (22.7)	
You are called names or insulted			P=.669
Never/rarely	266 (97.1)	21 (85.5)	
Sometimes/often/always	8 (2.9)	1 (4.6)	
You are threatened/harassed			P=.523
Never/rarely	269 (98.2)	22 (100.0)	
Sometimes/often/always	5 (1.8)	0	

* Participant reports they did not initiate of at least one component of therapy that was recommended to them by their treatment team (i.e., adiation, chemotherapy, hormonal therapy, trastuzumab).

+ Comparisons made using Chi-square tests; bolded results had P-value <.05.

## Data Availability

The datasets used and analyzed during the current study are available from the corresponding author on reasonable request.
